# Comparisons of Serum Interleukin-8 Levels in Major Depressive Patients With Drug-Free Versus SSRIs Versus Healthy Controls

**DOI:** 10.3389/fpsyt.2022.858675

**Published:** 2022-04-14

**Authors:** Zhen Hua Zhu, Xiao Ying Song, Li Juan Man, Peng Chen, Zhen Tang, Rong Hua Li, Cai Fang Ji, Ning Bin Dai, Fang Liu, Jing Wang, Jianping Zhang, Qiu Fang Jia, Li Hui

**Affiliations:** ^1^Research Center of Biological Psychiatry, Suzhou Guangji Hospital, Medical College of Soochow University, Suzhou, China; ^2^Suzhou Center for Disease Prevention and Control, Suzhou, China; ^3^Department of Psychiatry, Weill Cornell Medical College, Cornell University, New York, NY, United States

**Keywords:** major depressive disorder, interleukin-8, depressive symptom, SSRIs, association

## Abstract

**Objective:**

The interleukin-8 (IL-8) has been reported to play an important role in depression, which might be modulated by the selective serotonin reuptake inhibitors (SSRIs). Thus, the aim of this study was to investigate serum IL-8 levels, depressive symptom, and their associations in drug-free MDD patients, MDD patients with SSRIs, and healthy controls (HCs).

**Methods:**

Fifty-seven drug-free MDD patients (male/female = 35/22, mean age: 39.24 years), 30 MDD patients with SSRIs (male/female = 11/19, mean age: 39.73 years), and 101 HCs (male/female = 52/49, mean age: 37.38 years) were recruited in this cross-sectional study. Serum IL-8 levels and depressive symptom were assessed using the Flow Cytometer and Hamilton Depression Scale (HAMD). The analysis of variance was used for the comparison between groups. The relationship between serum log_10_ IL-8 levels and HAMD score was analyzed by Pearson correlation.

**Results:**

Serum log_10_IL-8 levels were lower in all patients than HCs after controlling for covariates (*F* = 4.86, *p* = 0.03). There was significant difference in serum Log_10_IL-8 levels among three groups after controlling for covariates (*F* = 14.63, *p* < 0.001). Serum Log_10_IL-8 levels in drug-free patients were lower compared to HCs (*F* = 19.38, *p* < 0.001) or patients with SSRIs (*F* = 21.89, *p* < 0.001) after controlling for covariates. However, there was not difference in serum log_10_IL-8 levels between patients with SSRIs and HCs after controlling for covariates. Moreover, serum Log_10_IL-8 levels were negatively correlated with HAMD score in all patients (*r* = −0.37, *p* = 0.02). Also, serum Log_10_IL-8 levels were negatively correlated with HAMD score in drug-free patients (*r* = −0.74, *p* = 0.01), but not in patients with SSRIs.

**Conclusion:**

Our data supported that the decline in serum IL-8 levels was association with depression. Moreover, the SSRIs might modulate increased serum IL-8 levels of depression.

## Introduction

Major depressive disorder (MDD) is a highly prevalent and severe psychiatric illness influencing psychological function and diminishing life quality (1). The clinical manifestations of MDD are one or more episodes of depressive mood, markedly diminished pleasure or interest, and even recurrent thoughts of death (1). In China, a larger cross-sectional epidemiological study has shown that the lifetime and 12-month prevalence of MDD are approximately 6.8 and 3.6%, respectively (2). In the United States, the lifetime and 12-month prevalence of MDD have been reported to be 20.6 and 10.4% in a sample of 36,309 adults, respectively (3). Globally, it is estimated that the lifetime and annual prevalence of MDD are 11.1 and 5.9%, respectively, in the 18 countries participating in the world mental health surveys (4). In addition, MDD is said to be one of the ten most disabling conditions worldwide (5, 6). The World Health Organization (WHO) has reported that by 2030, MDD will be the first leading cause of global disease burden (7). Thus, MDD has become an important global public health issue. However, the pathogenesis of MDD is still unclear and requires further investigation.

Neuroinflammation may be implicated in the underlying etiology of MDD, and it may be involved in the disruptions to neuroendocrine function, neurotransmitter metabolism, neuroplasticity, and neural circuity of MDD (8–11). The inflammation hypothesis for MDD is further supported by a serious of accumulating evidence, such as serum abnormal concentrations of inflammatory biomarkers (12–14), depression occurrence with the administration of endotoxin or vaccination (15–17), and the associations between aberrant inflammation cytokines and brain abnormalities of MDD (18, 19). Recently, increasing evidence from genetics studies have further indicated the pivotal role of neuroinflammatory cytokines in the MDD pathophysiology by detecting *C-reactive protein (CRP)* methylation, *IL-6* rs1800795, and *IL-1*β rs16944 as the risk loci of MDD (20–22).

Interleukin 8 (IL-8) is an important inflammatory cytokine synthesized and released mainly by macrophages, microglia and astrocytes (23, 24). Mounting evidence support MDD as a neuroinflammatory disorder that is closely related to abnormal activation of microglia and astrocytes (24–26). Thus, the association between IL-8 levels and MDD has be debated. For example, at the protein level, plasma/serum/cerebrospinal fluid (CSF) levels of IL-8 are significantly increased in patients with MDD in comparison to healthy controls (HCs) (27–31). At the molecular level, higher expression of the *IL-8* gene on chromosome 4q is reported in the prefrontal cortex of drug-free MDD (32). The rs4073 (–251T>A) polymorphism in the promotor region of *IL-8* gene is a functional locus that further influences the IL-8 production or protein expression both *in vivo* and vitro (33–36), and this polymorphism has been reported to be significantly associated with the susceptibility to depression (37, 38). However, previous studies also have indicated that the transcripts and translations for the *IL-8* gene show significant decline in patients with MDD compared with HCs (12, 39). In addition, several studies have reported that there are no significant differences in the protein and mRNA levels of IL-8 between patients with MDD and HCs (40–42). These discrepant results suggest that the role of IL-8 in depression should deserve further investigation. Moreover, several studies have indicated that the SSRIs might modulate IL-8 levels in patients with MDD and other psychiatric disorder (28, 43, 44).

However, to our best knowledge, few studies have examined the comparisons of serum IL-8 levels in drug-free MDD patients, MDD patients with SSRIs, and HCs in a Han Chinese population. Thus, the main aim of this study was to investigate serum IL-8 levels, depressive symptom, and their associations in three groups.

## Materials and Methods

### Ethics Statement

This study was conducted between August 2017 and May 2021. A research coordinator explained the study protocol and procedure to each participant, then signed informed consent was obtained. The study protocol and informed consent were approved by the Medical Institutional Review Board of Suzhou Guangji Hospital.

### Participants

Eighty-seven MDD patients (male/female = 46/41) including 57 drug-free MDD patients and 30 MDD patients with SSRIs (the administration of single SSRI) were enrolled from outpatient or inpatient unit of Suzhou Guangji Hospital, a municipal owned psychiatric hospital. The service area of this hospital covered approximately a population of 12.7 million people. All MDD patients met the following inclusion criteria: (1) Han Chinese, aged 18–60 years old; (2) confirmation of unipolar depression according to the Diagnostic and Statistical Manual of Mental Disorders, Fourth Edition (DSM-IV) by two experienced psychiatrists; (3) had a minimum of 6 years of education; and (4) had the ability to participate in the assessment of depressive scale. Moreover, MDD patient did not take any antidepressants, which was defined as drug-free patient. Patients with SSRIs also did not take other medication.

In total, 101 HCs (male/female = 52/49) were enrolled from the local community in the Suzhou Xiangcheng District. All HCs met the following inclusion criteria: (1) Han Chinese, aged 18–60 years; (2) received education for at least 6 years; (3) had the ability to participate in the assessment of depressive symptom; and (4) had a Zung Self-Rating Depression Scale (SDS) normal score < 50 that was assessed using the SDS Chinese version (45, 46). Current mental status and personal or family history of any mental disorder were assessed using unstructured interviews in HCs. In addition, none of HCs had any history of MDD.

All participants were in good physical health. Any participants with schizophrenia, schizoaffective disorders, dementia, neurodegenerative and neurological disorders, cardiovascular disease, cerebrovascular disease, infections, cancer, diabetes, hypertension, hyperlipidemia, and pregnancy were excluded.

### Clinical Measurements

A detailed questionnaire including a complete medical history, physical examination, and medical and psychological conditions was obtained from each participant. Additional information including age, gender, education, body mass index (BMI), smoking, drinking, and suicide status, age of onset was collected from available medical records.

Depressive symptom in all patients were assessed by using the Hamilton Rating Scale for Depression (24-items) (HAMD) (47–49). The consistency/reliability of HAMD (*p* < 0.01) has been established in MDD patients (50). The HAMD included a total of 24 items. The score range of 10 items was from 0 to 2 that represented: 0 = none, 1 = mild-moderate, and 2 = severe, respectively. In addition, the score range of 14 items was from 0 to 4 that represented: 0 = none, 1 = mild, 2 = moderate, 3 = severe, and 4 = very severe, respectively (50). The measurement of HAMD was conducted by a research coordinator after the patient recruitment.

### Interleukin-8 Measurement

Blood samples with coagulants were collected from all participants between 7 and 9 AM following an overnight fasting. The serum was separated, aliquoted, and stored at −80°C in a refrigerator before laboratory assays. Serum IL-8 levels were measured by the BD™ FACSCanto Flow Cytometer and BD™ Cytometric Bead Array (CBA) Human Inflammatory Cytokines Kit (BD Biosciences, San Jose, CA, United States). The detailed experiment procedure was conducted according to the instruction manuals of the kit and the flow cytometer. The sensitivity for IL-8 measurement was 3.6 pg/ml in this assay, with 4% intra- and inter- assay variation coefficients, respectively. A IL-8 standard curve was established from the BD™ CBA Human Inflammatory Cytokine Standards in triplicate for the experiment per batch. This experiment was conducted by the same technician who was blind to the clinical information and sample’s identity document.

### Statistical Analysis

The demographic and clinical data were compared between all MDD patients and HCs using an analysis of variance (ANOVA) for continuous variables and a Chi-squared test for categorical variables. Serum IL-8 levels were not normally distributed and were thus log-transformed. We then compared serum log_10_IL-8 levels between all MDD patients and HCs using an ANOVA. When the significance was found in the ANOVA, the potential confounding factors (sex, age, education, BMI, smoking, drinking and suicide status) were added as covariates. Serum log_10_IL-8 levels were further compared among drug-free MDD patients, MDD patients with SSRIs, and HCs using the ANOVA and analysis of covariance (ANCOVA), respectively. *Post-hoc* comparisons between groups were made using the Fisher’s least significant difference (LSD) procedure. In addition, the comparisons of serum log_10_IL-8 levels were analyzed in drug-free MDD patients versus HCs, MDD patients with SSRIs versus HCs, and drug-free MDD patients versus MDD patients with SSRIs using the ANCOVA. According to sex grouping, the comparisons of serum log_10_IL-8 levels were analyzed among drug-free MDD patients, MDD patients with SSRIs and HCs using the ANCOVA. Moreover, the comparisons of serum log_10_IL-8 levels were analyzed among suicide attempters with drug-free MDD, non-suicide attempters with drug-free MDD and non-suicide attempters of HCs as well as among suicide attempters with SSRI-medicated MDD, non-suicide attempter with SSRI-medicated MDD and non-suicide attempters of HCs using the ANCOVA. The correlations between serum log_10_IL-8 levels and HAMD score in drug-free MDD patients, MDD patients with SSRIs, and all MDD patients were evaluated with Pearson’s product moment correction coefficients, respectively. Continuous data were presented as the mean and standard deviation (mean ± SD), and all *p*-values were 2-tailed at a significance level of <0.05.

## Results

### Demographic and Clinical Characteristics

There were no significant differences in sex, age, BMI, smoking, and drinking between all MDD patients and HCs (Table 1). However, education (*F* = 58.02, *p* < 0.001) and suicide status (χ^2^ = 56.51, *p* < 0.001) were significantly different between two groups. The mean and SD of age of onset (years), and HAMD score in all MDD patients were 34.69 ± 13.44, and 21.38 ± 6.97, respectively. A total of 87 MDD patients include 57 drug-free MDD patients (65.5%) and 30 MDD patients with SSRIs (34.5%). Moreover, education and suicide status were significantly different in HCs versus drug-free patients versus patients with SSIRs (*F* = 29.92, *p* < 0.001; χ^2^ = 56.65, *p* < 0.001), HCs versus drug-free patients (*F* = 55.36, *p* < 0.001; χ^2^ = 47.46, *p* < 0.001), and HCs versus patients with SSRIs (*F* = 21.87, *p* < 0.001; χ^2^ = 42.19, *p* < 0.001). Sex (*F* = 3.89, *p* = 0.04) was significantly different in drug-free patients versus patients with SSRIs. However, the HAMD score (*F* = 0.98, *p* = 0.33) was not different in drug-free patients versus patients with SSRIs.

**TABLE 1:** Demographic and clinical data between all major depressive disorder (MDD) patients and healthy control (HCs).

Variables	All MDD patients	HCs	F or χ ^2^	*p*
	*N* = 87, Mean (SD)	*N* = 101, Mean (SD)		
Gender (male/female)	46/41	52/49	0.002	0.97
Age (years)	39.40 (13.79)	37.38 (12.13)	0.29	0.44
Education (years)	9.47 (3.45)	13.00 (2.90)	58.02	<0.001^**^
BMI (kg/m^2^)	22.40(3.57)	22.94 (3.03)	1.22	0.27
Smoking (smoker/non-smoker)	71/16	76/25	0.57	0.45
Drinking (drinker/non-drinker)	71/16	92/9	2.87	0.09
Suicide (attempters/non-attempters)	50/37	7/94	56.51	<0.001^**^
Age of onset (years)	35.69 (13.44)	–	–	–
HAMD score	21.38 (6.97)	–	–	–
Type of antidepressants				
No treatment	57 (65.5%)	–	–	–
Single SSRI	30 (34.5%)	–	–	–

MDD, major depressive disorder; HCs, healthy controls; SD, standard deviation; SSRIs, selective serotonin reuptake inhibitors; BMI, body mass index; HAMD, Hamilton Depression Scale. **p < 0.01.

### Comparisons of Serum Log_10_IL-8 Levels

**FIGURE 1:**
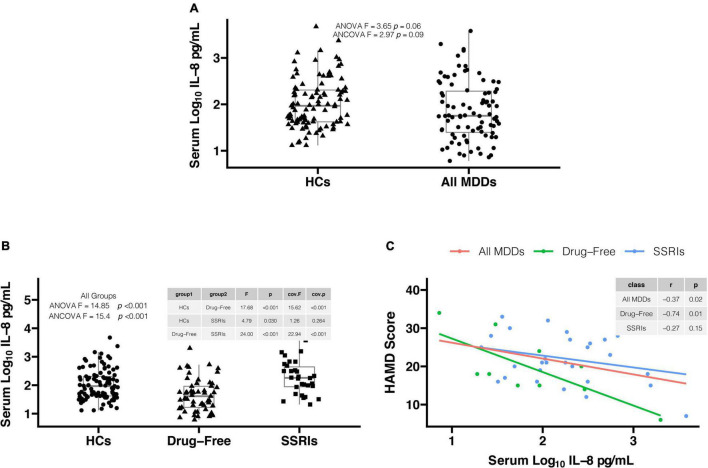
**(A)** Serum log_10_IL-8 levels in all major depressive disorder (MDD) patients were lower in comparison to healthy controls (HCs) after adjusting for covariates (1.86 ± 0.64 vs. 2.03 ± 0.53, *F* = 4.86, *p* = 0.03). **(B)** There was significant difference in serum log_10_IL-8 levels among three groups after adjusting for covariates (1.65 ± 0.56 vs. 2.27 ± 0.57 vs. 2.03 ± 0.53, *F* = 14.63, *p* < 0.001). Serum log_10_IL-8 levels were significantly lower in drug-free MDD patients in comparison to MDD patients with SSRIs (*F* = 21.89, *p* < 0.001) and HCs (*F* = 19.38, *p* < 0.001) after adjusting for covariates. However, there was no difference in serum log_10_IL-8 levels between MDD patients with SSRIs and HCs after adjusting for covariates (*F* = 3.68, *p* > 0.05). **(C)** The significant correlations were found in drug-free MDD patients (*r* = –0.74, *p* = 0.01) and all MDD patients (*r* = –0.37, *p* = 0.02), but not in MDD patients with SSRIs (*r* = –0.27, *p* = 0.15). IL-8, interleukin-8; MDD, major depressive disorder; HCs, healthy controls; SSRIs, selective serotonin reuptake inhibitors; HAMD, Hamilton Depression Scale; ANOVA, analysis of variance; ANCOVA, analysis of covariance.

There was not significant difference in serum log_10_IL-8 levels between all MDD patients and HCs in Figure 1A (1.86 ± 0.64 versus 2.03 ± 0.53, *F* = 3.65, *p* = 0.06). The significant difference was observed between two groups after adjusting for sex, age, education, BMI, smoking, drinking and suicide status in Figure 1A (*F* = 4.86, *p* = 0.03). The confounding factors including education and suicide status did not directly influence the comparative index of serum log_10_IL-8 levels between two groups (all, *p* > 0.05). There was significant difference in serum log_10_IL-8 levels among drug-free MDD patients, MDD patients with SSRIs, and HCs in Figure 1B (1.65 ± 0.56 versus 2.27 ± 0.57 versus 2.03 ± 0.53, *F* = 14.85, *p* < 0.001). This difference was still significant after adjusting for covariates (*F* = 14.63, *p* < 0.001). Sex (*F* = 4.90, *p* = 0.03) and age (*F* = 4.71, *p* = 0.03) of covariates directly influenced the comparative index of serum log_10_IL-8 levels among three groups. Further *post-hoc* comparisons found the significant differences in serum IL-8 levels in drug-free MDD patients versus HCs (*F* = 17.68, *p* < 0.001), MDD patients with SSRIs versus HCs (*F* = 4.79, *p* = 0.03), and drug-free MDD patients versus MDD patients with SSRIs (*F* = 24.00, *p* < 0.001) using Fisher’s LSD procedure in Figure 1B. The significant differences in drug-free MDD patients versus HCs (*F* = 19.38, *p* < 0.001), and drug-free MDD patients versus MDD patients with SSRIs (*F* = 21.89, *p* < 0.001) were still observed, but not in MDD patients with SSRIs versus HCs (*F* = 3.68, *p* > 0.05) after adjusting for covariates in Figure 1B. The effect of sex on the comparative index of serum log_10_ IL-8 levels in drug-free patients versus HCs (*F* = 5.44, *P* = 0.02), and patients with SSRIs versus HCs (*F* = 4.35, *p* = 0.04) was observed as well as the effect of age on the comparative index of serum log_10_ IL-8 levels in patients with drug-free versus SSRIs (*F* = 4.91, *P* = 0.03).

In male, there were significant differences in serum log_10_IL-8 levels in drug-free patients versus patients with SSRIs versus HCs (*F* = 7.45, *p* = 0.001), drug-free patients versus HCs (*F* = 12.39, *p* < 0.001), and drug-free patients versus patients with SSRIs (*F* = 8.97, *p* = 0.005) rather than patients with SSRIs versus HCs (*F* = 0.43, *p* = 0.52) after adjusting for covariates. In female, there were significant differences in serum log_10_IL-8 levels in drug-free patients versus patients with SSRIs versus HCs (*F* = 9.53, *p* < 0.001), drug-free patients versus HCs (*F* = 8.19, *p* = 0.006), patients with SSRIs versus HCs (*F* = 7.68, *p* = 0.007), and drug-free patients versus patients with SSRIs (*F* = 12.20, *p* = 0.001) after adjusting for covariates. However, there were no significant differences in serum log_10_IL-8 levels between male and female in HCs (*p* > 0.05), drug-free patients (*p* > 0.05), and patients with SSRIs (*p* > 0.05) after adjusting for covariates.

In addition, there was significant difference in serum log_10_IL-8 levels in suicide attempters with drug-free MDD versus non-suicide attempters with drug-free MDD versus non-suicide attempters of HCs after adjusting for covariates (*F* = 9.29, *p* = 0.003). Compared to non-suicide attempters of HCs, serum log_10_IL-8 levels were significantly lower in suicide attempters with drug-free MDD (*F* = 14.76, *p* < 0.001) and non-suicide attempters with drug-free MDD (*F* = 8.67, *p* = 0.004) after adjusting for covariates. In addition, serum log_10_IL-8 levels in suicide attempters with SSRI-medicated MDD were significantly lower than those in non-suicide attempters of HCs after adjusting for covariates (*F* = 4.01, *p* < 0.05).

### Correlations Between Serum Log_10_IL-8 Levels and Hamilton Depression Scale Score

In Figure 1C, the Pearson correlation analysis showed that serum log_10_IL-8 levels were significantly correlated with HAMD score in all MDD patients (*r* = −0.37, *p* = 0.02) and drug-free MDD patients (*r* = −0.74, *p* = 0.01), but not in MDD patients with SSRIs (*r* = −0.27, *p* = 0.15).

## Discussion

To our best knowledge, it is the first cross-sectional study to investigate serum IL-8 levels, depressive symptom, and their associations in drug-free MDD patients, MDD patients with SSRIs, and HCs in a Han Chinese population. Two main findings were listed as follows: (1) serum log_10_IL-8 levels were significantly lower in drug-free MDD patients and all MDD patients in comparison to HCs, and serum log_10_IL-8 levels were negatively associated with depressive symptom score in all MDD patients and drug-free MDD patients, respectively; (2) serum log_10_IL-8 levels in MDD patients with SSRIs were higher than those in drug-free MDD patients, but did not show in patients with SSRIs and HCs.

Accumulating evidence have indicated that the abnormalities of neuroinflammation might be implicated in the pathogenesis of MDD. IL-8 has been regarded as a crucial inflammatory cytokine produced by macrophages, as well as brain cells such as microglia and astrocytes (23, 24). Thus, the IL-8 abnormality might play an important role in the pathogenesis of MDD. Our data has shown significant decline in serum log_10_IL-8 levels in all MDD patients and drug-free MDD patients in comparison to HCs, and serum log_10_IL-8 levels were negatively correlated with HAMD score in all MDD patients and drug-free MDD patients, respectively. The potential mechanism responsible for the association between decreased serum IL-8 levels and depression was that the decline in IL-8 levels might reflect the occurrences of altered microglia-mediated synaptic pruning and/or reduced astrocytes in the brain of MDD (51–56). Previous studies have suggested that the decline in serum IL-8 levels might be involved in the depression. For example, a recent meta-analysis showed that peripheral IL-8 levels were significantly decreased in drug-free first-episode MDD patients in comparison to HCs (12). Another meta-analysis study of small sample including two studies has found that CSF IL-8 levels were significantly lower in 38 MDD patients compared to 114 HCs (31). A study of gene expression profiling in the dorsolateral prefrontal cortex of brain has reported that the transcripts of *IL-8* gene were significantly decreased in MDD patients with suicides and non-suicides in comparison to HCs (39), suggesting that the decline in *IL-8* expression was implicated in depression neurobiology. In addition, serum IL-8 levels were reported to be inversely related to depressive symptom score in individuals with astrocytoma (57). The above reports supported a notion that the decline in serum IL-8 levels played a vital role in depression. However, previous studies also have found that the transcript and translation levels of *IL-8* were significantly higher in MDD patients in comparison to HCs (27–32). Moreover, several studies also reported no significant differences in the IL-8 protein and mRNA levels between MDD patients and HCs (40–42). These discrepant results of the IL-8 comparative index in patients with MDD versus HCs should deserve to be further investigated in future study.

Moreover, previous studies have indicated the effects of different inflammatory processes on different subtypes of depression (58–60). For example, non-melancholic patients exhibited the cytokine production of increased pro-inflammatory, whereas melancholic patients, in contrast, exhibited the cytokine production of reduced pro-inflammatory (61–63). The decline in IL-8 expression in the dorsolateral prefrontal cortex has been reported to be involved in the neurobiology of MDD suicides (39). The dorsolateral prefrontal cortex has been found to be implicated in the regulation of impulsivity, decision-making, cognitive control of mood and other executive function related to suicidal behavior (64, 65), and functional imaging studies further supported the deficits in perfusion and cortical thickness in prefrontal cortex of suicides before death and suicide attempters (66, 67). The data of current study has also shown that the ratio of suicide attempters in all MDD patients was 57.47%, and decreased serum IL-8 levels were observed in suicide attempters with drug-free MDD, non-suicide attempters with drug-free MDD, and suicide attempters with SSRIs-medicated MDD in comparison to non-suicide attempters of HCs, respectively. Thus, suicide attempters with MDD should be regarded as an important subtype that could influence the results of current study based on the relationship between IL-8 and suicidal ideation (68).

Interestingly, our study found that serum IL-8 levels were significantly elevated in MDD patients with SSRIs compared with drug-free MDD patients, but not in MDD patients with SSRIs versus HCs. This result suggested that the SSRIs might modulate increased serum IL-8 levels in patients with MDD. The SSRIs have been reported to modulate microglia and astrocytes in hippocampus to produce the inflammatory cytokines including IL-8 (69–73). Moreover, MDD has been considered a neuroinflammatory disorder that was implicated in the abnormal activities of brain microglia and astrocytes (24–26). A previous study has demonstrated that the monotherapy with escitalopram was found to elevate the IL-8 trend of depression, which further alleviated depressive symptom (74). The above findings supported the notion that the SSRIs were implicated in modulating the microglia and astrocytes, which caused increased IL-8 level of depression. Moreover, two previous studies have found that the electroconvulsive therapy (ECT) treatment might significantly increase serum/CSF IL-8 levels in patients with MDD (75, 76). These findings further suggested that increased IL-8 levels in MDD patients reduced depressive symptom, which further hinted that increased IL-8 levels might have neuroprotective effects for MDD (77, 78). However, the SSRIs were reported to cause the decline in plasma IL-8 levels in patients with MDD (43). In addition, the SSRIs were found to decrease serum IL-8 levels in patients with generalized anxiety disorder (44). These inconsistent findings suggested that future study of a large and independent sample should be carried out to confirm the present findings in first-episode drug-free patients with MDD.

Although previous studies have reported that sex differences influenced inflammatory processes of depression (79–81), the present study did not find the effect of sex differences on serum IL-8 levels in drug-free patients or patients with SSRIs. However, our data showed that serum IL-8 levels in female SSRI-medicated MDD patients were significantly higher than those in female HCs, but did not show in male SSRIs-medicated MDD patients versus male HCs, which further indicated that female might be especially sensitive to the effect of SSRIs on serum IL-8 levels of depression.

The present study had several limitations as follows: (1) A relatively small sample size. The effect size of present study was calculated to be very small (η2 = 0.02). Thus, our results should be regarded as a pilot study. (2) Employing banked samples that were collected from August 2017 to May 2021, and were stored at –80°C in a refrigerator for differing time lengths. The storage time might influence the IL-8 measurement in this study; (3) A cross-sectional design. Thus, it was unclear whether there were causative relationships among serum IL-8 levels, HAMD score, and SSRIs in MDD patients. Thus, further studies with longitudinal and prospective follow-up were necessary to clarify the time course of potential SSRIs effects on IL-8 levels in patients with MDD. (4) Confirmed diagnosis of MDD. Although the patients were confirmed with a diagnosis for unipolar depression rather than bipolar depression at the moment of patient recruitment, a few unipolar depressive patients might convert to bipolar depressive patients during follow-ups. (5) *MDD classification*. Serum IL-8 levels might be influenced by MDD classification such as first-episode/chronic/recurrence/recovery patients. (6) *SSRI types and doses*. The effects of different SSRI types and doses on IL-8 levels also had discrepant in patients with MDD (43). (7) Depressive symptom was assessed using the Zung Self-Rating Depression Scale in healthy controls and the Hamilton Depression Scale in participants with MDD. This limited the correlation analysis between depressive symptom severity and IL-8 levels to those with confirmed MDD. (8) the absence of other clinical data and assessments including diet, sleep, and duration of illness. Future studies should be expanded to include those data that might influence serum IL-8 levels and depressive symptom of MDD. Finally, (9) IL-8 levels were analyzed in serum, it is unknown whether the decline in serum IL-8 levels may reflect the decline in IL-8 levels in the brain of MDD in comparison to HCs due to the blood-brain barrier, which should further deserve to be investigated. However, the mRNA expression of *IL-8* gene in the dorsolateral prefrontal cortex of depression has been reported to be significantly lower in comparison to HCs (39).

Moreover, the present study had several strengths as follows: (1) It was first cross-sectional study to investigate serum IL-8 levels in drug-free MDD patients versus MDD patients with SSRIs versus HCs in a Han Chinese population. (2) The sensitivity for IL-8 measurement was 3.6 pg/ml in this assay with 4% intra- and inter- assay variation coefficients, which further supported the credibility of present results.

In summary, serum IL-8 levels were lower in all MDD patients and drug-free MDD patients in comparison to HCs, and serum IL-8 levels were negatively correlated with depressive symptom in drug-free MDD patients and all MDD patients. These data supported that the decline in serum IL-8 levels contributed to depression. Serum IL-8 levels were higher in MDD patients with SSRIs in comparison to drug-free MDD patients, but not in MDD patients with SSRIs versus HCs. These data further demonstrated that the SSRIs might modulate increased serum IL-8 levels of MDD. However, the current findings still were preliminary because of the relatively small sample size and absence of a longitudinal follow-up. Therefore, future studies were warranted to verify the current findings in a large and independent cohort of first-episode drug-free patients with MDD.

## Data Availability Statement

The raw data supporting the conclusions of this article will be made available by the authors, without undue reservation.

## Ethics Statement

The studies involving human participants were reviewed and approved by the Medical Institutional Review Board of Suzhou Guangji Hospital. The patients/participants provided their written informed consent to participate in this study.

## Author Contributions

ZZ, XS, LM, and LH were responsible for the study design, statistical analysis, and manuscript preparation. ZZ, XS, LM, PC, ZT, RL, CJ, JW, and QJ were responsible for recruiting the patients, performing the clinical rating, and collecting the samples. ND and FL were involved in evolving the ideas and editing the manuscript. QJ and LH were involved in writing the protocol, providing the funding for the study, and editing the manuscript. JZ commented on the manuscript critically. All authors have contributed and approved the final manuscript.

## Conflict of Interest

The authors declare that the research was conducted in the absence of any commercial or financial relationships that could be construed as a potential conflict of interest.

## Publisher’s Note

All claims expressed in this article are solely those of the authors and do not necessarily represent those of their affiliated organizations, or those of the publisher, the editors and the reviewers. Any product that may be evaluated in this article, or claim that may be made by its manufacturer, is not guaranteed or endorsed by the publisher.
