# Supplementary material for: Comparisons of Serum Interleukin-8 Levels in Major Depressive Patients With Drug-Free Versus SSRIs Versus Healthy Controls
Source: Front Psychiatry. 2022 Apr 14;13:858675. doi: 10.3389/fpsyt.2022.858675 (PMC9046727; doi:10.3389/fpsyt.2022.858675)
Supplement: Supplementary file 1 [file Data_Sheet_1.pdf]

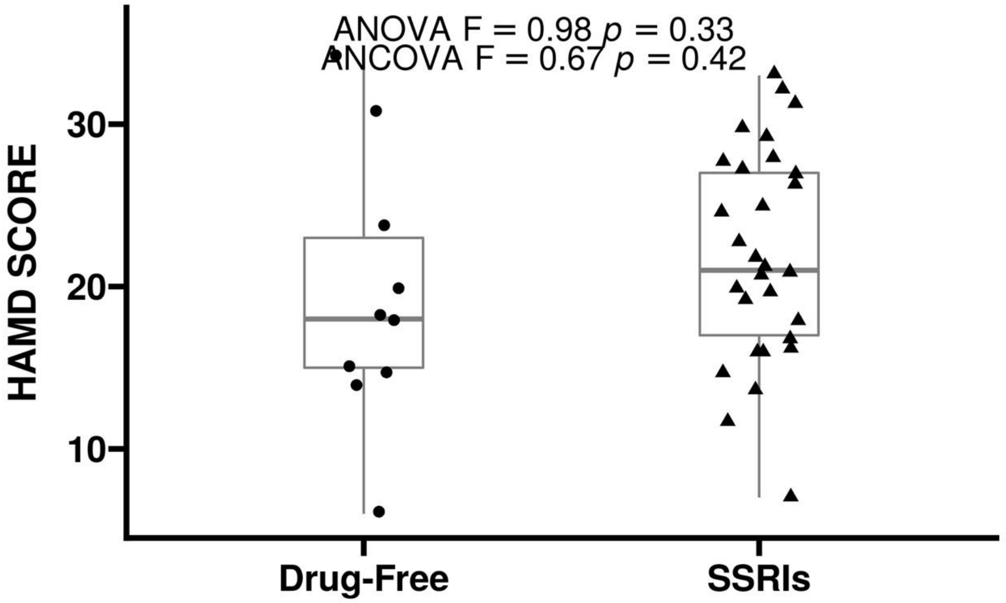

Supplementary Fig. 1. The HAMD score (F = 0.98, p = 0.33) was not different in MDD patients with drug-free versus SSRIs.

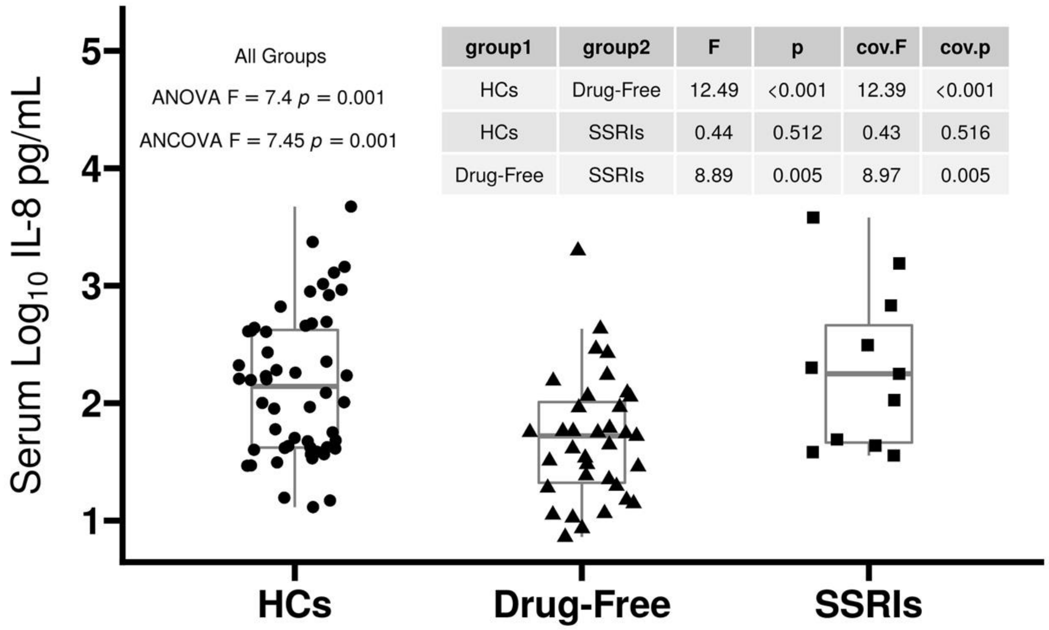

Supplementary Fig. 2. The comprasions of serum $\log_{10}$ IL-8 levels among MDD patients with drug-free versus SSRIs versus HCs in male.

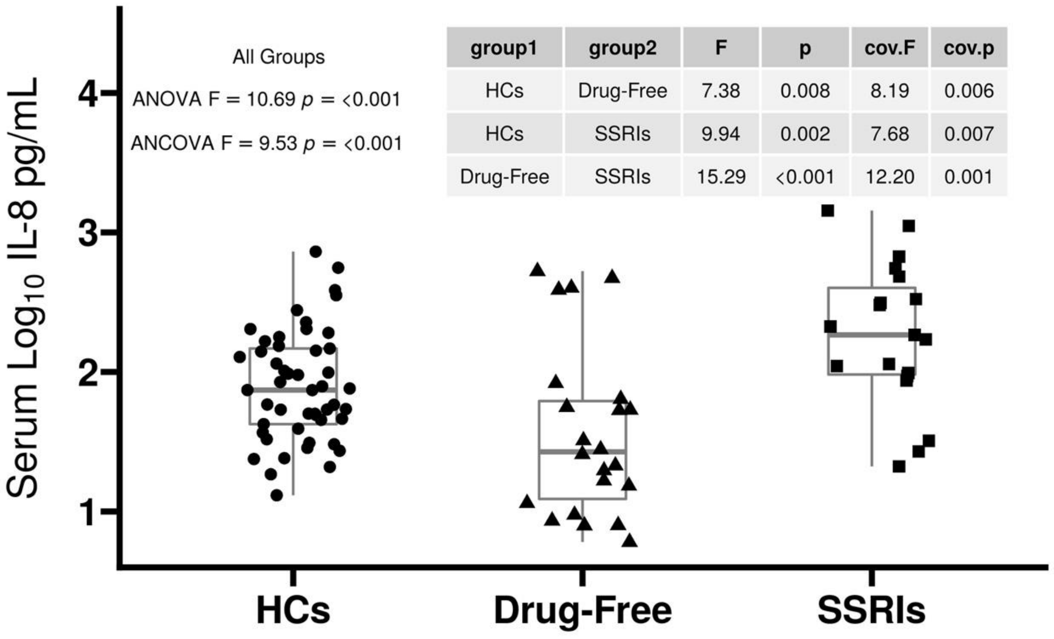

Supplementary Fig. 3. The comprasions of serum $\log_{10}$ IL-8 levels among MDD patients with drug-free versus SSRIs versus HCs in female.

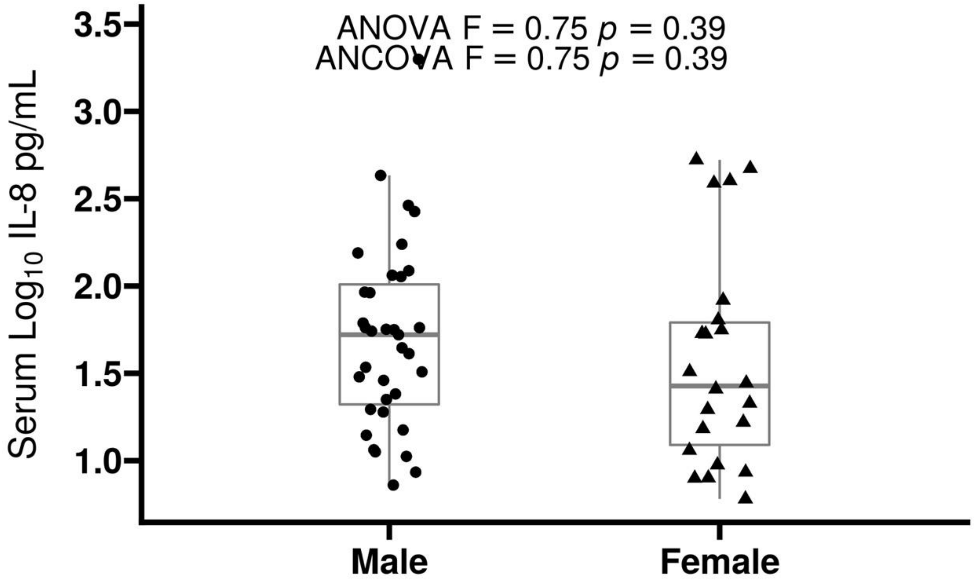

Supplementary Fig. 4. The comprasion of serum $\log_{10}$ IL-8 levels between male and female in MDD patients with drug-free.

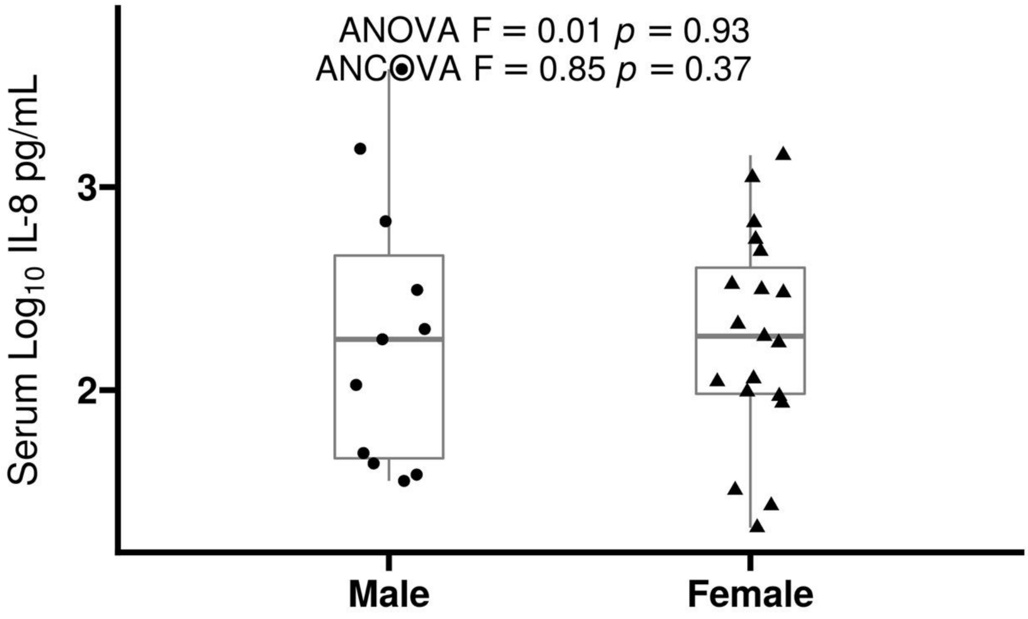

Supplementary Fig. 5. The comprasion of serum $\log_{10}$ IL-8 levels between male and female in MDD patients with SSRIs.

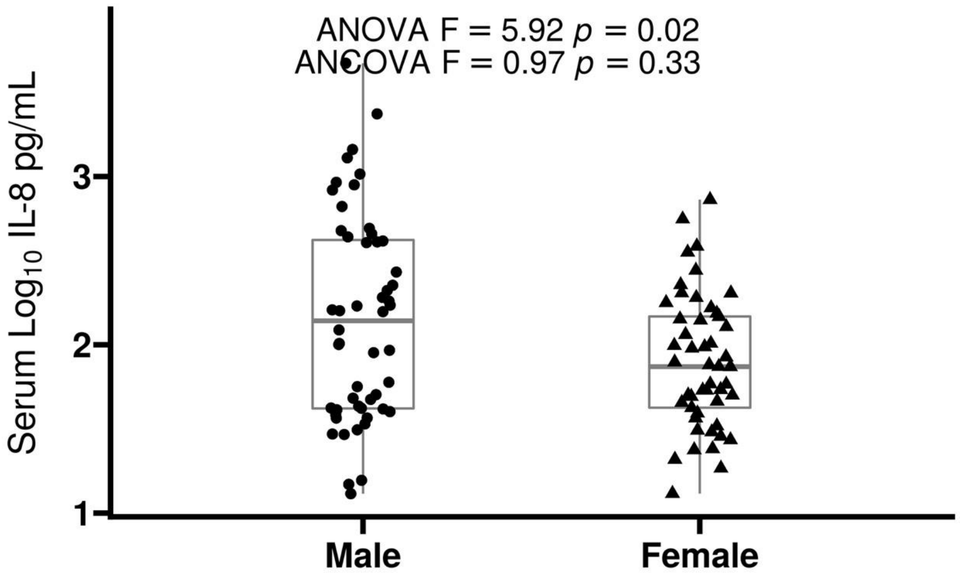

Supplementary Fig. 6. The comprasion of serum $\log_{10}$ IL-8 levels between male and female in HCs.

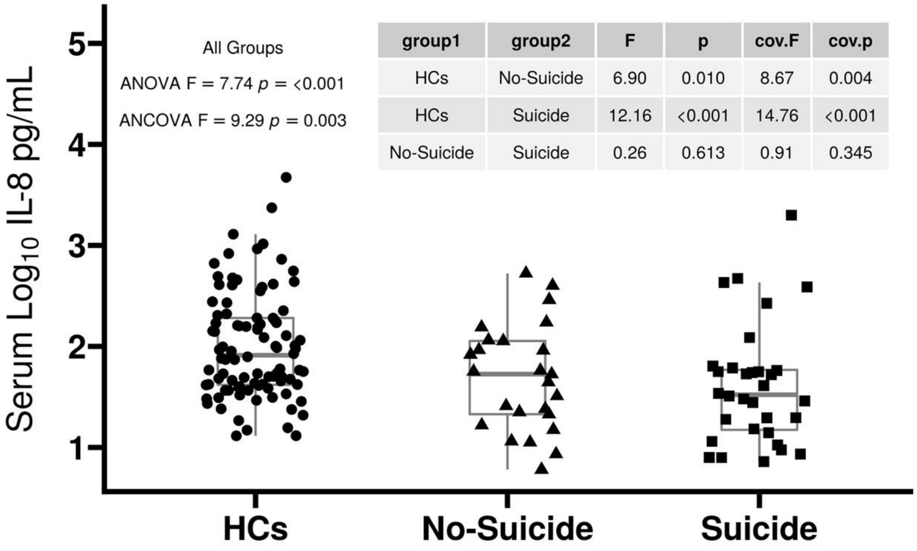

Supplementary Fig. 7. The comprasions of serum $\log_{10}$ IL-8 levels among suicide attempters with drug-free MDD, non-suicide attempters with drug-free MDD and non-suicide attempters of HCs.

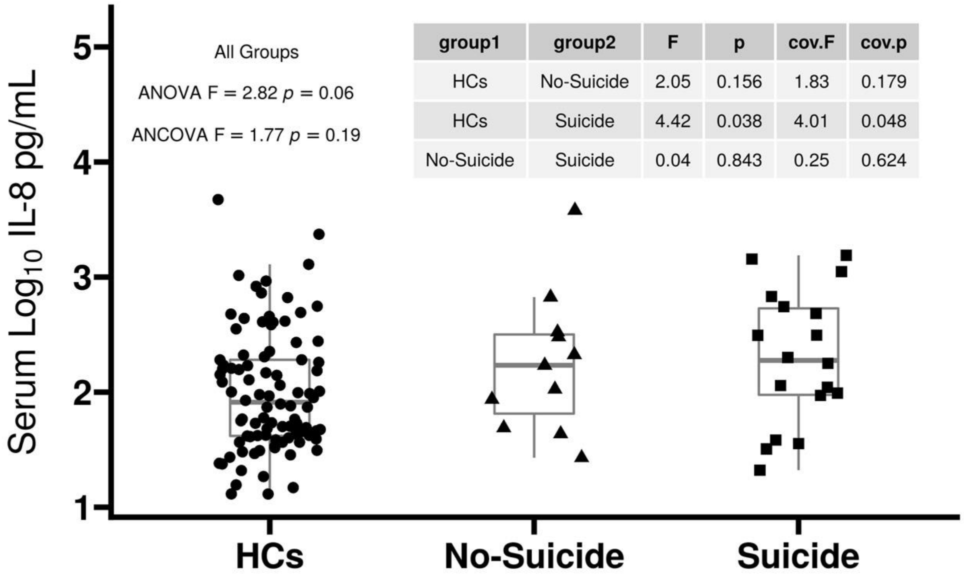

Supplementary Fig. 8. The comprasions of serum $\log_{10}$ IL-8 levels among suicide attempters with SSRI-medicated MDD, non-suicide attempters with SSRI-mediated MDD and non-suicide attempters of HCs.
